# Progress of Science

**Published:** 1841-11

**Authors:** 


### PROGRESS OF SCIENCE.

It must gratify the friends of sound and solid science to learn, that the *College of Professional Teachers,* at their late meeting in our sister city of Cincinnati, requested *doctor* Rosenstein, the famous homœopathist, to enlighten them and the good people of that city, with a lecture. Such a thirst for knowledge is highly auspicious. We presume the *doctor* administered to each member of the College, the true homœopathic dose, of a hundred thousandth part of a grain of truth, mingled in a glass of gall and wormwood against the *regular* profession. But why should the College have limited itself to the homœopathists, when the Steam doctors, Negro doctors, Indian doctors, Brandreth doctors, and others were at hand, and ready to expound their respective systems? Such partiality we cannot approve; it is at war with the true democracy of science, and, if persisted in, will undoubtedly bring the College into disfavour with the friends of those improved systems, which are more numerous than the advocates of homœopathy. In this way the College will render itself unpopular and diminish its influence. Perhaps, however, it has appointed committees to make reports at the next meeting, on those reformed methods; and thus obviated the charge of partiality. If so, very well; but lest it should not have regarded any of the other new improvements as worthy of encouragement, (after contemplating the beauties of homœopathy) we take the liberty of presenting the first rude draft of a preamble and resolutions, to be adopted at the next annual meeting of the College.

Whereas, in the opinion of this College, established to promote sound learning and exact science, the world has been held in bondage for three thousand years, by a succession of men calling themselves the disciples of Hippocrates, who profess to have made discoveries concerning the laws of the human body and the means of preventing and curing its diseases, whereby they have imposed on the unwary, to the great detriment of their constitutions; and whereas a number of original geniuses, in modern times, have made important hygienic discoveries, which are rejected by the aforesaid disciples of Hippocrates, through jealousy and envy of said discoverers, and from malice prepense towards society; and whereas it is the duty of this College to foster neglected genius, not less than to humble the pride of science, therefore—

*Resolved*, That a committee of seven members, not physicians, be appointed to make a report, to the next session of the College, in favour of the following newly discovered systems of medicine for the people, towit:

1. On Hanemannism, or Homœopathic doctoring;
2. On Mesmerism, or Magnetic doctoring;
3. On Cornstalkism, or Indian doctoring;
4. On Radicalism, or Root doctoring;
5. On Brandrethism, or Pill doctoring;
6. On Thompsonianism, or Steam doctoring;
7. On Guineaism, or Negro doctoring.

*Resolved*, That said committee be instructed, to warn the community against the pretended discoveries of Hippocrates, Celsus, Morgani, Harvey, Sydenham, Bichat, Rush, and others of like kind, and against the schools of medicine in the United States, where their writings are received as authority.

*Resolved,* That the members of this College will henceforth, patronize some one of the aforesaid seven wise men; preferring, however, the last, from the well known curative powers of a seventh son.

*Resolved,* That should any other *College of Professional Teachers,* attempt to forestall this, in the proposed reform of the medical profession, it shall be the duty of the "Executive Committee" to appoint a special meeting of the College, that it may, by early action, not lose the honor of achieving what it is the first to undertake.

*Resolved,* That in all future meetings of the College, the practisers of the aforesaid seven new systems, shall be entitled to seats; and that such of them as cannot write their names, shall be allowed to subscribe the constitution by making their marks. D.

